# Safety and efficacy of sertraline in depression among adults undergoing dialysis: a systematic review and meta-analysis

**DOI:** 10.1097/MS9.0000000000002677

**Published:** 2024-10-22

**Authors:** Asfia Qammar, Bazil Azeem, Sateesh Kumar, Madhurta Kumari, Farhad Hassan, Laiba Khurram, Sumet Kumar, Abdul Fasih, Arwa Khan, Muhammad Basit Azeem, Nimra Sadiq, Ramsha Dibaj, Varsha Sharma

**Affiliations:** a Baylor Scott & White Heart and Vascular Hospital, Dallas, TX, USA; b Shaheed Mohtarma Benazir Bhutto Medical College Lyari, Karachi, Pakistan; c Chandka Medical College, SMBBMU, Larkana, Pakistan; d Liaquat University of Medical and Health Sciences, Jamshoro, Pakistan; e Department of Internal Medicine, Nepal Medical College, Gokarneshwar, Nepal

**Keywords:** adults, depression, dialysis, sertraline, SSRI

## Abstract

**Background:**

Depression is prevalent among patients with end-stage renal disease (ESRD) undergoing dialysis, with significant implications for their quality of life and treatment compliance. Traditional treatments for depression, including various therapies and pharmacological interventions, have limitations due to their adverse effects. Sertraline, a selective serotonin re-uptake inhibitor (SSRI), offers a promising alternative, but its efficacy and safety in this population require thorough evaluation.

**Objective:**

This meta-analysis aims to assess the effectiveness and adverse effects of sertraline in treating depressive episodes in dialysis patients compared to placebo.

**Methods:**

Following the Preferred Reporting Items for Systematic Reviews and Meta-Analyses (PRISMA) guidelines, the authors conducted a comprehensive search of databases, including PubMed, Cochrane Library, and Science Direct, up to 20 June 2024. The authors included randomized controlled trials (RCTs) that compared sertraline with placebo in dialysis patients with depression. Two researchers independently performed data extraction and risk of bias assessment. Statistical analysis was conducted using ReviewManager 5.4.1, employing a random effects model.

**Results:**

Four RCTs involving 468 participants were included. Sertraline significantly reduced depressive symptoms, as measured by the Quick Inventory of Depressive Symptomatology (QIDS) and Beck Depression Inventory-II (BDI-II) scores, at 6 and 12 weeks compared to placebo. Improvements in kidney disease-specific quality of life (KDQOL-36) scores were also noted. However, sertraline was associated with a higher risk of adverse events compared to placebo.

**Conclusions:**

Sertraline effectively reduces depressive symptoms and improves the quality of life in dialysis patients with ESRD. Despite the increased risk of adverse events, the overall benefits make sertraline a viable treatment option for this population. Larger, more comprehensive studies are needed to confirm these findings and optimize sertraline use in clinical practice.

## Introduction

Highlights

- Depression, highly prevalent in dialysis patients, affects treatment outcomes and survival rates; traditional treatments are often limited by adverse effects and interactions with other medications.
- Results showed that sertraline significantly reduced depressive symptoms at both 6 and 12 weeks compared to placebo, with improvements measured by the QIDS and BDI-II scores.
- Sertraline also led to significant improvements in quality of life, as indicated by KDQOL-36 scores, although it was associated with a higher incidence of adverse events compared to placebo.
- Adverse events associated with sertraline were generally mild to moderate and manageable, making it a viable treatment option for depression in dialysis patients.
- This meta-analysis highlights the importance of addressing depression in dialysis patients and suggests that sertraline can be an effective treatment, though careful monitoring for adverse events is necessary.

Depression is the predominant mood disorder among patients with end-stage renal disease who undergo dialysis^1^, is a pressing issue. The epidemiological stats about depression reveal a prevalence of 23.7% in patients with chronic renal disease, among which 34.5% of patients are undergoing dialysis^2^. This mood disorder further exacerbates the severity of CKD by disrupting the quality of life and compliance of patients toward the treatment^3^. Common psychiatric conditions include depression, anxiety, dementia, delirium, and attention deficit hyperactivity disorder (ADHD)^4,5^. These disorders can negatively impact patients’ prognosis, quality of life, and mortality rates^6,7,8^. This underscores the need for timely intervention in treating depression associated with haemodialysis patients.

While there are several traditional treatments available for depression, including non-pharmacological interventions for depressive symptoms in ESRD patients, psychological interventions, exercise programs, and manual acupressure, which have shown promising results in alleviating symptoms (Li *et al.* 2023; Wen *et al.* 2020), cognitive behavioural therapy and exercise training are specifically mentioned as treatment options. However, the effectiveness of these non-pharmacological approaches is limited by the small scale and short duration of existing studies^9^. Also, certain pharmacological treatments, e.g., Tricyclic inhibitors and non-selective serotonin re-uptake inhibitors, usage might decline due to the numerous adverse effects such as electrolyte imbalance, sedation, cognitive impairment, etc^10^. The altered drug clearance in ESRD patients poses challenges for medication use^11^. These effects, both mild and severe, could decrease their usage, necessitating the exploration of alternative solutions.

Serotonin is a mood regulator neurotransmitter that plays a crucial role in mitigating the symptoms of depression in the central nervous system; it is supported by many types of research that have shown a decline in the number of serotonin metabolites in the spinal fluid of depressive patients^12^. Sertraline, an SSRI, has shown promising results in managing psychological distress in haemodialysis patients. Although side effects are possible, they are typically mild. They can be controlled and reversed by splitting the dosage, lowering the amount of a single dose or multiple doses, or altering the time when medications are taken^13^.

This meta-analysis is of utmost importance as it aims to evaluate the effectiveness and adverse effects related to the use of sertraline for treating depressive episodes in patients with renal diseases undergoing dialysis in comparison with placebo. The findings of this analysis, which could significantly impact the treatment of depression in this patient population, will provide valuable insights and contribute to the advancement of nephrology and psychiatry.

## Materials and methods

The Preferred Reporting Items for Systematic Reviews and Meta-Analyses (PRISMA) criteria, a set of guidelines for reporting systematic reviews and meta-analyses, and assessment of multiple systemic reviews (AMSTAR) 2, were both followed in conducting this systematic review and meta-analysis. Also, this meta-analysis was registered on PROSPERO (CRD42024563162). These criteria are designed to ensure transparency and completeness in reporting systematic reviews, which is crucial for the reliability and reproducibility of research findings. In our study, adherence to the PRISMA criteria ensures that our review is comprehensive, transparent, and methodologically sound, thereby enhancing the trustworthiness of our findings^14^.

### Data sources and search strategy

We conducted an extensive search of the MEDLINE database via PubMed, the Cochrane Library, and Science Direct up to 20 June 2024. Additionally, online databases such as www.clinicaltrials.gov were also searched to identify grey literature. The search strategy was created using MeSH terms and keywords related to sertraline, haemodialysis, depression, and depressive disorder. The specific MeSH terms and keywords used in the search included “sertraline,” “hemodialysis,” “depression,” “depressive disorder,” “RCT,” and “placebo.” Boolean operators like “AND,” “OR,” and “NOT” were employed to refine the search and ensure comprehensive coverage. We used a combination of these terms and Boolean operators to ensure a comprehensive search. No language restrictions were applied during the search process to ensure a broad inclusion of studies. Two researchers carried out each search procedure separately. The precise search technique, including the specific terms used and the Boolean operators employed, is shown in **supplementary table 1A**, http://links.lww.com/MS9/A631

### Study selection

After extracting every article from the databases, we moved it to EndNote X9 (ClarivateTM) in order to eliminate duplicates. Independently, two investigators in the group reviewed the titles and abstracts of the potentially relevant publications before moving on to reading the complete texts. Every step of the screening procedure was recorded. Inclusion criteria for randomised controlled trials (RCTs) were: (1) Participants were dialysis patients diagnosed with depression; (2) Subjects were 18 years of age or older; (3) The intervention consisted of sertraline with a placebo control group for comparison; and (4) Studies reported at least one of the outcomes of interest (QIDS, BDI-II, KDQOL-36, or TEAEs). Exclusion criteria included studies that did not meet the inclusion criteria, non-RCT studies, and studies that did not provide sufficient data for extraction. The primary studies were ultimately identified from four randomized controlled trials (RCTs) that were analyzed (Hedayati *et al.*, 2017; Mehrotra *et al.*, 2019; Friedli *et al.*, 2017; Zhang *et al.*, 2024)^15–18^. There were no language-based exclusions in the selection of randomized control studies.

### Data extraction

Two researchers extracted the data from the included articles independently using a self-created information extraction form. Discrepancies were solved by discussion or consulting a third researcher. The study name and year of publication, the study design, the mean age of patients in each group, the number of patients in each group, hypertensive and diabetic patients, hemoglobin level, and all outcomes of interest were collected from each study. The primary outcome was the quick inventory of the total score of depressive symptomatology (QIDS) between the sertraline and placebo groups from the baseline to the last visit. Secondary outcomes were changes in Beck depression inventory (BDI-II) scores, kidney disease quality of life (KDQOL-36) scores, and treatment-emergent adverse events (TEAEs).

### Risk of bias assessment

We evaluated the risk of bias in the included RCTs using the Risk of Bias 2 tool (RoB 2) as advised by the Cochrane Collaboration^19^. The RoB 2 tool evaluates five domains: the randomisation process, deviation from intended intervention, missing outcome data, measurement of the outcome, and selection of the reported result. Also, outcome assessment and selection bias within published results were all considered while evaluating the research. Each study was thoroughly examined before being assigned a bias risk rating of “low risk,” “unclear risk,” or “high risk.” Two independent reviewers carried out the RoB assessment and settled any disagreements. In case of disagreement, a third reviewer was consulted to reach a consensus.

### Statistical analysis

We used ReviewManager (RevMan Version 5.4.1) (Cochrane *et al.*, UK) to perform the statistical analysis. Risk ratios (RR) were used to portray dichotomous outcomes, while the mean difference (MD) and standard deviation (SD) were used to present continuous outcomes. A random effects model was used. The 95% CI was calculated for each effect size estimate. The heterogeneity among the included studies was presented using Higgins’s I^2^ measure^20^, where a value of I^2^ less than 50% was considered mild heterogeneity, a value between 50 and 75% was regarded as moderate heterogeneity, and a value over 75% was considered severe heterogeneity. The *P* less than 0.05 or equivalent was considered statistically significant.

## Results

### Study selection and characteristics

**Figure 1:**
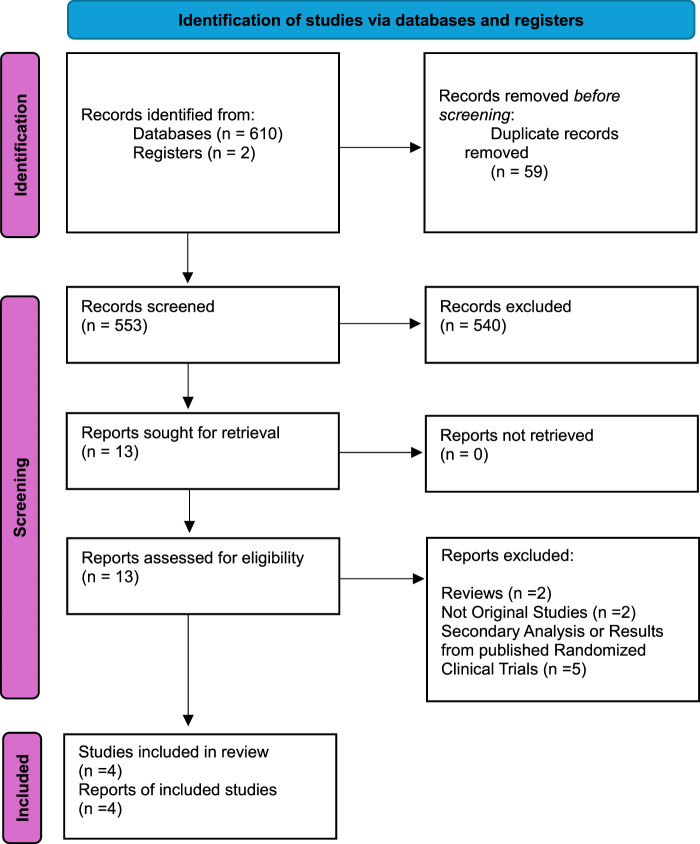
Preferred Reporting Items for Systematic Reviews and Meta-Analyses (PRISMA) flowchart.

Our study selection process was rigorous and thorough. Initially, we identified 612 studies from various databases, including PubMed, Cochrane Library, clincaltrial.gov, and Science Direct. After eliminating 59 duplicate records, we meticulously screened 553 studies for eligibility. Following an in-depth examination, 540 studies were excluded, and 13 reports were checked for retrieval. All 13 reports were retrieved and further scrutinized for eligibility. According to our strict inclusion criteria, only four studies were deemed eligible for our meta-analysis. The flowchart of the PRISMA statement, a widely accepted tool for systematic reviews and meta-analyses, below clearly summarizes our meticulous screening process (Fig. 1). The included four studies reported data on a total of 468 participants. Randomized control trials were conducted in different areas: two in the United States, one in the United Kingdom, and one in China. The mean age of participants is 58 in the sertraline group and 56.2 in the placebo group. A summary of included studies and patient baseline characteristics is given in the table (Tables 1 and 2).

**Table 1:** General characteristics of included studies table.

					Patients					
Study name	Study year	Country	Trial identifier no.	Total sample size	Sertraline	Control group	Primary outcome	Drug dose (mg)	Follow-up duration (week)	Patient category
Hedayati *et al.* ^15^	2017	USA	NCT00946998	193	97	96	Quick inventory of depressive symptomatology	50–200 mg	12	Haemodialysis patients with depressive disorder
Mehrotra *et al.* ^16^	2019	USA	NCT02358343	120	60	60	Quick inventory of depressive symptomatology	25–50 mg	12	Haemodialysis patients with depressive disorder
Friedli *et al.* ^17^	2017	UK	ISRCTN06146268	30	15	15	Quick inventory of depressive symptomatology	50 mg	NA	Haemodialysis patients with depressive disorder
Zhang *et al.* ^18^	2024	China	NCT06124417	125	62	63	Quick inventory of depressive symptomatology	25–50 mg	12	Haemodialysis patients with depressive disorder

**Table 2:** Patient baseline characteristics table.

		Patients		Age—year		Sex M/F		Hypertension		Diabetes mellitus		Hemoglobin, mean (SD), g/dl		QIDS Score at baseline		BDI-II At baseline	
Study name	Total sample size	Sertraline	Control group	Sertraline	Control group	Sertraline	Control group	Sertraline	Control group	Sertraline	Control group	Sertraline	Control group	Sertraline	Control group	Sertraline	Control group
Hedayati *et al.* ^15^	193	97	96	57.7 (14.5)	59.1 (12.2)	74/23	67/29	94	94	58	54	11.9 (2.1)	12.0 (2.2)	14.0 (2.4)	14.1 (2.4)	NA	NA
Mehrotra *et al.* ^16^	120	60	60	53±12	50±13	35/25	33/27	54	55	38	35	11.1±1.3	10.8±1.5	10.9 (4.9)	12.2 (5.1)	25.8 (8.7)	26.2 (10)
Friedli *et al.* ^17^	30	15	15	61.7 (13.2)	56.4 (14.4)	11;4	12;3	NA	NA	6	7	11.8 (1.9)	11.7 (1.4)	NA	NA	24.5 (4.5)	25.3 (4.2)
Zhang *et al.* ^18^	125	62	63	59.63±13.00	59.22±15.43	31;31	35;28	52	56	17	20	10.9±1.52	10.7 (1.98)	NA	NA	NA	NA

NA, not applicable.

### Risk of bias of included studies

**Figure 2:**
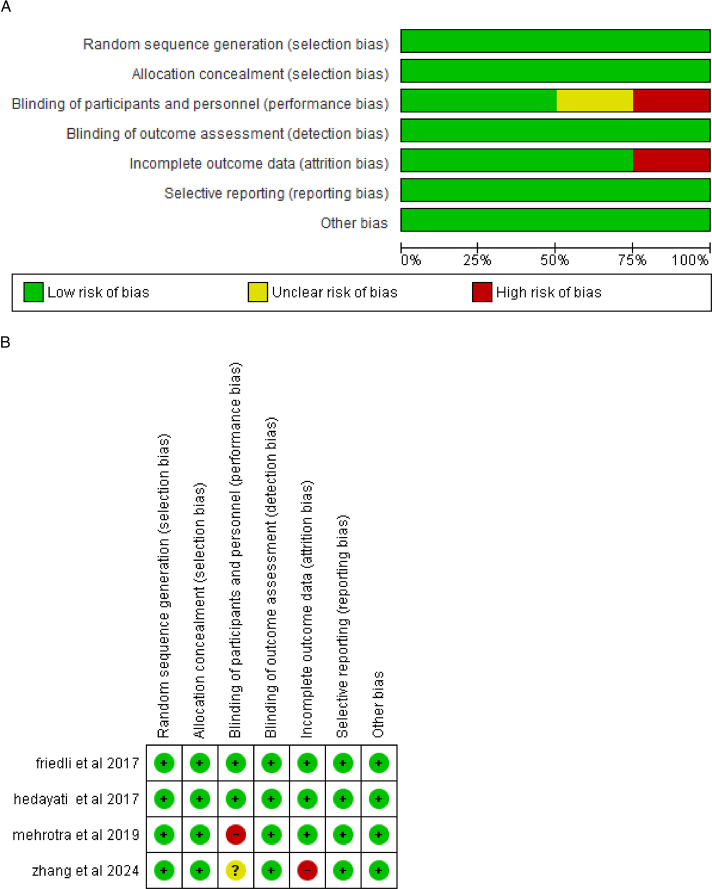
(A) Risk of bias graph. (B) Risk of bias summary.

Our assessment of the risk of bias was conducted in accordance with the Cochrane Handbook for systematic reviews and meta-analysis, ensuring a standardized and objective approach. All the randomized control trials (RCTs) included in our meta-analysis demonstrated a low overall risk of bias. However, we identified specific concerns in the studies of Mehrotra and colleagues and Zhang and colleagues^16,18^. In these studies, particularly in the areas of blinding participants (performance bias) and blinding of outcome assessment (detection bias), we observed potential issues that could affect the reliability of the results. These concerns are detailed in Figure 2A, B and Supplemental Table 2, http://links.lww.com/MS9/A631.

### Primary outcomes

#### Changes from baseline in QIDS score

**Figure 3:**
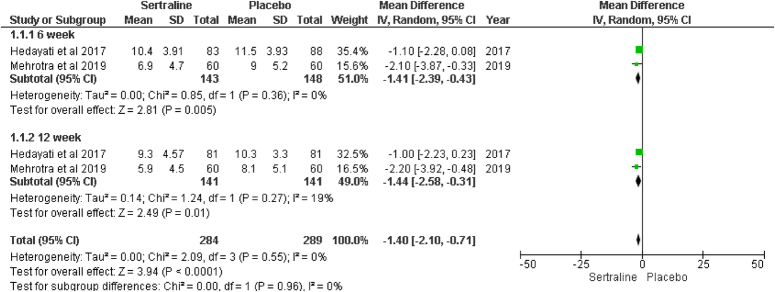
Forest plot of change from baseline in Quick Inventory of Depressive Symptomatology (QIDS) Score.

Quick Inventory of Depressive Symptomatology (QIDS) Score is assessed at 6 and 12 weeks as the primary outcome; two studies, that is Hedayati and colleagues and Mehrotra and colleagues^15,16^, This outcome was reported during these specified periods (Fig. 3).

#### Changes from baseline in QIDS score at 6 weeks

Both studies, Hedayati and colleagues and Mehrotra and colleagues^15,16^, Indicate a significant reduction in QIDS score at 6 weeks with sertraline treatment (MD: −1.41; 95% CI: −2.39, −0.43; *P*=0.0005; I^2^=0%). This finding underscores the potential of sertraline in effectively reducing depressive symptoms.

#### Changes from baseline in QIDS score at 12 weeks

The effectiveness of sertraline was also better than the placebo at 12 weeks in reducing the QIDS score significantly. However, some concerns about heterogenicity were there, but they turned out to be non-significant (MD: −1.44; 95% CI: −2.58, −0.31; *P*=0.01; I^2^=19%).

#### Overall effect

The sertraline group was associated with low QIDS scores compared to the placebo group (MD: −1.40; 95% CI: −2.10, −0.71; *P* <0.0001; I^2^=0%).

### Secondary outcomes

Three secondary outcomes were analyzed after being categorized into subgroups. These include a depression score, scores for overall kidney health with physical and mental assessment in kidney patients, and adverse drug effects, which are mentioned below with their respective subgroups.

#### Changes from baseline in BDI-II score

**Figure 4:**
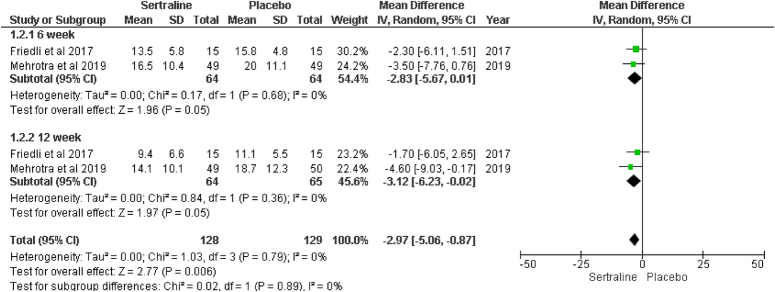
Forest plot of change from baseline in Beck Depression Inventory-II (BDI-II) Score.

Beck Depression Inventory-II (BDI-II), a widely used 21-item self-reported inventory, is assessed to measure the severity of depression in participants. Two studies, Friedli and colleagues and Mehrotra and colleagues^16,17^ (Fig. 4), reported this at 6 and 12 weeks.

#### Changes from baseline in BDI-II score at 6 weeks

This outcome is reported in two studies and suggests the efficacy of the sertraline group was associated with low BDI-II score after 6 weeks when compared to the placebo group (MD: −2.83; 95% CI: −5.67, 0.01; *P*e=0.05; I^2^=0%).

#### Changes from baseline in BDI-II score at 12 weeks

Participants in the sertraline group were again associated with low BDI-II even after 12 weeks than the placebo group, elucidating the efficacy of sertraline (MD: −3.12, 95% CI: −6.23 to −0.02; *P*=0.05; I^2^=0%).

#### Overall effect

The sertraline group is associated with low BDI-II score and hence proved to be profoundly effective in treating depression in dialysis patients compared to the placebo group during analysis (MD: −2.97; 95% CI: −5.06, −0.87; *P*=0.006; I^2^=0%).

#### Changes from baseline in KDQOL-36 score

**Figure 5:**
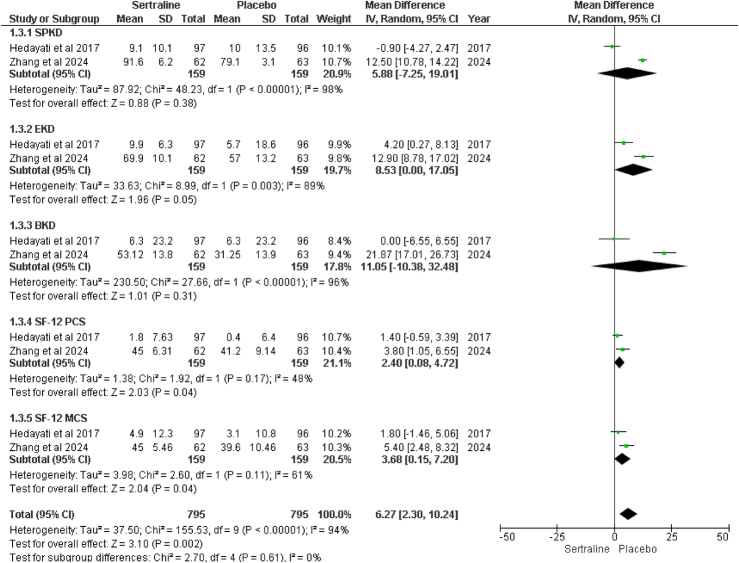
Forest plot of change from baseline in kidney disease-specific quality of life (KDQOL-36) Scores.

The kidney disease quality of life-36 score is used to assess the quality of life in renal patients. The assessment is further divided into five subgroups to analyze the efficacy of sertraline better. In two studies, Hedayati and colleagues and Zhang and colleagues^15,18^, reported these outcomes (Fig. 5).

#### Changes from baseline in SPKD score

The analysis indicates that the sertraline group showed a significant improvement in the symptoms and problems associated with kidney disease compared to the placebo group. However, considerable group heterogenicity is observed in this variable of the KDQOL-36 Score (MD: 5.88; CI: −7.25 to 19.01; *P*=0.38; I^2^=98%). This heterogenicity was addressed by performing subgroup analyses and sensitivity analyses to explore the impact of different study characteristics on the outcomes.

#### Changes from baseline in EKD

With sertraline treatment, the effects of kidney disease on daily life improve statistically, though the effect is at the significance threshold. Again, high heterogeneity indicates variable effects across different patient demographics or study conditions (MD: 8.53; 95% CI: 0.00, 17.05; *P*=0.05; I^2^=89%).

#### Changes from baseline in BKD

Sertraline noticeably improved the perceived burden of kidney disease, suggesting that the results might vary more among the population (MD: 11.05; 95% CI: −10.38, 32.48; *P*=0.31; I^2^=96%).

#### Changes from baseline in SF-12 PCS

Sertraline leads to a statistically significant improvement in the physical health components of quality of life (MD 2.40; 95% CI: 0.08, 4.72; *P*=0.04) with moderate heterogeneity I^2^=48% which was non-significant, *P* value=0.17, indicating more consistency in this outcome.

#### Changes from baseline in SF-12 MCS

There is a statistically significant improvement in mental health components with sertraline, indicating better emotional well-being compared to placebo (MD: 3.68; 95% CI: 0.15, 7.20; *P*=0.04) with moderate heterogenicity I^2^=61%, which was non-significant, *P* value=0.11.

#### Overall effect

This plot depicted that sertraline can be beneficial in improving both the specific symptoms associated with kidney disease and patients’ overall physical and mental health. However, the variability in effects, with high heterogeneity (MD: 6.27; 95% CI: 2.30, 10.24; *P*=0.002; I^2^=94%). The heterogeneity observed was addressed by conducting sensitivity analyses, which involved removing one study at a time to assess the impact on overall results and considering the study settings and population characteristics.

#### Treatment-emergent adverse events (TEAEs)

**Figure 6:**
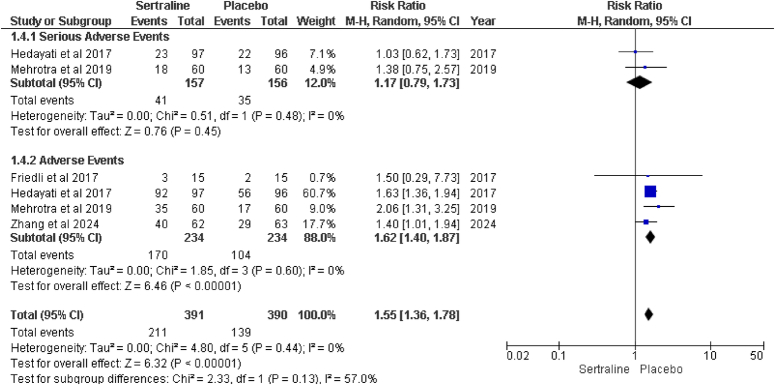
Forest plot of treatment-emergent adverse events (TEAEs).

TEAEs are assessed for the safety of sertraline on patients, so two subgroup analyses were performed for adverse and serious adverse events (Fig. 6).

#### Adverse events

All four studies^15–18^ Reported adverse events in their studies. The most common adverse events reported included nausea, dizziness, insomnia and fatigue. The pooled RR for adverse events was 1.62 (95% CI: 1.40–1.87), suggesting a statistically significant increase in the risk of adverse events for patients taking sertraline compared to those taking placebo (*P*<0.00001). However, it is important to note that no heterogeneity was observed among these studies (I²=0%, *P*=0.60).

#### Serious adverse events

Two studies^15,16^ Included the report of serious adverse events associated with sertraline compared to placebo. Serious adverse events were less frequent and primarily involved cardiovascular events and severe gastrointestinal issues. The pooled RR for serious adverse events was 1.17 (95% CI: 0.79–1.73), indicating no statistically significant difference between sertraline and placebo (*P*=0.45). The heterogeneity among these studies was low (I²=0%, *P*=0.48).

#### Overall adverse events

Combining both serious and non-serious adverse events across the studies, the total RR was 1.55 (95% CI: 1.36–1.78), indicating a significantly higher risk of adverse events for sertraline compared to placebo (*P*<0.00001). The heterogeneity for this combined analysis was also low (I²=0%, *P*=0.44).

## Discussion

We performed a meta-analysis examining the effects of sertraline on patients having dialysis. Our study is an original meta-analysis that offers specific insights into the subject matter. We included four randomized controlled trials (RCTs) in our analysis, which investigated various primary and secondary outcomes. Our primary endpoint was QIDS at 6 and 12 weeks, and we also examined secondary endpoints such as Beck Depression Inventory-II at 6 and 12 weeks and various parameters of KDQOL-36 score; we also assessed the adverse events at the end of the study. Our findings indicate that both the primary and secondary outcomes were statistically significant. Overall, our results suggest that sertraline is beneficial compared to other treatments in renal patients having dialysis. However, it is important to exercise caution when interpreting these findings, as they may only be reliable with further investigation. We urge the medical community to join us in this endeavour, as our study provides information on the benefits of sertraline in reducing depressive symptoms and improving symptoms and problems associated with kidney disease, the effects of kidney disease on daily life, the perceived burden of kidney disease, physical health components, and mental health components of quality of life.

Depression is a prevalent comorbidity in chronic disease populations, necessitating effective and comprehensive treatment strategies^21^. Current treatment options encompass a range of psychological and pharmacological interventions. Psychotherapy, particularly cognitive-behavioural therapy (CBT), is often the first line of treatment for mild to moderate depression, offering significant benefits without the risk of medication side effects^22^. For more severe cases, antidepressant medications such as selective serotonin re-uptake inhibitors (SSRIs), including fluoxetine and sertraline, serotonin-norepinephrine re-uptake inhibitors (SNRIs) like venlafaxine, and tricyclic antidepressants (TCAs) are widely used^23^. Additionally, electroconvulsive therapy (ECT) and repetitive transcranial magnetic stimulation (rTMS) are considered for treatment-resistant depression, demonstrating efficacy in patients unresponsive to conventional therapies^24^. In the context of renal disease patients undergoing dialysis, managing depression poses particular challenges^24^. The pharmacokinetics of many antidepressants can be altered in these patients due to compromised renal function, leading to concerns about drug accumulation and potential toxicity^25^. While non-pharmacological treatments such as psychotherapy and lifestyle modifications are beneficial, they may not be sufficient to address severe depressive symptoms^26^. Despite the availability of multiple treatment modalities, the management of depression in dialysis patients remains suboptimal^27^. Many antidepressants carry risks of side effects, including gastrointestinal disturbances, sexual dysfunction, and cardiovascular issues, which can be exacerbated in patients with chronic kidney disease (CKD)^28^. Additionally, the efficacy of these treatments can be variable, with some patients showing little to no improvement, underscoring the need for new, more effective treatment options that are well-tolerated and safe for patients with renal impairment^29^. One promising option is sertraline, a selective serotonin re-uptake inhibitor (SSRI) (Hedayati *et al.* 2016). Sertraline inhibits serotonin re-uptake in the brain, thereby increasing the availability of this neurotransmitter and enhancing mood. Its favourable side-effect profile and relatively straightforward pharmacokinetics make it a suitable candidate for treating depression^30^. Moreover, sertraline has been found to reduce systemic inflammation, a common issue in dialysis patients, which may contribute to its efficacy in alleviating depressive symptoms. This anti-inflammatory effect could further benefit patients by addressing both mood symptoms and inflammation-related complications common in dialysis patients^31^. This is particularly relevant as inflammation plays a role in depression pathogenesis in chronic kidney disease patients. This may explain the working of sertraline to reduce depression in dialysis patients. Given the complexities and challenges associated with managing depression in this population, sertraline offers a potential solution that warrants further exploration and consideration.

Various studies have explored the impact of sertraline on reducing depressive symptoms in patients with depressive disorders. Research has found that sertraline is linked to reduced depressive symptoms and enhances mood and quality of life^32^. Although the study findings were limited to a variety of depressive patients. However, other studies have revealed that sertraline also reduces anxiety, eating disorders, post-menstrual dysphoric disorder, and substance abuse^33^. This benefit is not limited to patients with depressive disorders, but it also extends to those with co-existing diabetes or chronic kidney disease^34^. These results align with the findings of our study, which examined the effect of sertraline on depressive symptoms in renal disease patients having dialysis. Additionally, research has shown that Sertraline use is associated with a reduction in depressive symptoms, underscoring the potential of sertraline in effectively reducing depressive symptoms in dialysis patients^18^. This aligns with findings from Hedayati and colleagues and Mehrotra and colleagues^15,16^, who reported similar outcomes. This is due to its ability to inhibit the presynaptic re-uptake of serotonin, leading to increased postsynaptic receptor response. However, previous meta-analyses, such as those by Palmer *et al.* ^35^, presented mixed results. Also, studies such as Bautovich *et al.* ^36^ often reported less pronounced improvements with other antidepressants, suggesting that sertraline might have a superior efficacy profile for dialysis patients. It has also been observed that sertraline improves the KDQOL-36 score in people with renal disease having dialysis, leading to an increased quality of life^18^. However, the complete mechanism is unknown. The outcomes of this study were consistent with the results of previous research. For instance, Rapaport reported similar improvements in patients with posttraumatic stress disorder, and Turner also noted positive changes in quality of life domains in patients with major depression^32,37^. This benefit was more noticeable in patients with severe heart failure^38^. However, Hedayati 2017 did not find a significant improvement in KDQOL-36 scores in non-dialysis-dependent CKD patients. Sertraline, a selective serotonin re-uptake inhibitor (SSRI), has been shown to improve both the physical and mental components of health in dialysis patients. Studies have demonstrated its efficacy in alleviating depressive symptoms, improving quality of life, and increasing treatment compliance^17,18^ It has also been found to reduce systemic inflammation, a common issue in dialysis patients, and improve depression symptoms^31^ as discussed earlier.

Sertraline, a commonly used SSRI, has been associated with a range of adverse effects in chronic renal patients; studies found adverse effects regardless of dialysis dependence^13^. However, patients having dialysis have many adverse effects, including gastrointestinal, central nervous system, and skin disorders^13,39^. These effects are likely due to its mechanism of action, which involves the inhibition of serotonin re-uptake into presynaptic terminals. This can lead to increased levels of serotonin in the gut, which may contribute to gastrointestinal side effects such as nausea, vomiting, and diarrhoea^40^. The drug’s impact on the central nervous system can also lead to nervous system disorders, while its influence on the skin and subcutaneous tissues can result in skin-related issues^41^. Compared to other SSRIs with higher QT-prolonging potential (citalopram, escitalopram), sertraline and other SSRIs with lower QT-prolonging potential were associated with a lower risk of sudden cardiac death in haemodialysis patients^42^. A systematic review found evidence favoring sertraline over other antidepressants in terms of efficacy (compared to fluoxetine) and acceptability/tolerability (compared to amitriptyline, imipramine, paroxetine, and mirtazapine)^43^. Sertraline has been shown to improve both the physical and mental components of health in dialysis patients^44^. It has also been found to reduce systemic inflammation, a common issue in dialysis patients, and improve depression symptoms^31^. These studies support our results, which show that sertraline is associated with more adverse events. Research suggests that individualized treatment plans for depression in dialysis patients could benefit from considering various factors. Pharmacogenomic testing can predict sertraline metabolism and guide medication selection in depressive patients^45^. However, further research is needed to fully understand the specific mechanisms by which sertraline causes these adverse effects and discuss the individualized treatment plan in detail. Also, to standardize the criteria for assessing adverse events, a consensus-driven framework in collaboration with regulatory agencies and clinical experts is needed, which should include uniform definitions, grading scales, and reporting guidelines for adverse events. In terms of cost-effectiveness, selective serotonin re-uptake inhibitors (SSRIs) are more cost-effective than tricyclic antidepressants (TCAs) for treating depression when considering overall healthcare utilization and expenses; sertraline, along with paroxetine, has lower acquisition costs among established SSRIs and is available in extended dosage forms to reduce multi tablet therapy^46^.

Heterogeneity was observed in one of our endpoints, changes from baseline in KDQOL-36 scores, showing high and significant heterogeneity (I²>75%) in the subdomains of symptoms/problems and the effects of kidney disease on daily life. This heterogeneity is mainly due to the results from Zhang *et al.* ^18^. This variability indicates different effects across patient demographics or study conditions. It could stem from differences in baseline characteristics, concomitant medications, or variations in dialysis protocols across studies. The differences in the population studied by Zhang *et al.* ^18^. Moreover, their findings likely contributed to this heterogeneity. This underscores the need for individualized treatment approaches and highlights the complexity of managing depression in dialysis patients. Studies with variations in dialysis protocols, including subgroup analyses and differences in frequency and type of dialysis, are also needed to understand how these factors contribute to heterogenicity.

### Limitations

Although our study was of high quality, it had some drawbacks. The quality of our research may have been affected by the small sample size. In addition, we could not perform a meta-regression because of the shortage of studies. The quality of our investigation may have been improved using this approach. Third, we had to compare various medications because of the shortage of data. This may have further enhanced the effects of medication. Another drawback of our study was that we were unable to discuss the cost-effectiveness of our medication, and the assessment of adverse events was based on varying criteria across studies, potentially biasing the safety profile of sertraline. These parameters could create a significant impact on this drug profile. Moreover, the reliance on self-reported measures for depressive symptoms may introduce subjective bias. Also, the included RCTs did not provide a detailed breakdown of the adverse events associated with sertraline use, such as the frequency and severity of side effects like nausea, dizziness, insomnia, and cardiovascular events. This limits the ability to understand the safety profile of sertraline in dialysis patients fully. Furthermore, the RCTs lacked sufficient subgroup analyses based on factors such as age, sex, and the duration of dialysis. Such subgroup analyses could provide more personalized insights into the benefits and risks of sertraline across different patient populations.

Additionally, the small number of included trials and the heterogeneity across studies in terms of study design, patient characteristics, and outcome measurements restrict the generalizability of our findings. The variability observed, particularly in quality-of-life outcomes, suggests the need for more standardized reporting and methodology in future studies.

### Clinical implication

The clinical implication of these findings suggests that sertraline could be integrated into treatment guidelines as a preferred option for managing depression in dialysis patients, given its efficacy and safety profile. The improved quality of life and reduced depressive symptoms observed with sertraline use could translate into better patient outcomes, including increased adherence to dialysis protocols and overall treatment regimens. These benefits may also reduce hospitalization rates and improve long-term survival. However, balancing these benefits with the potential risk of adverse events remains crucial, emphasizing the need for individualized patient assessments and monitoring.

This meta-analysis supports the efficacy of sertraline in reducing depressive symptoms and improving the quality of life in dialysis patients with ESRD. However, the increased risk of adverse events and the lack of detailed subgroup analyses in the included RCTs warrant caution. Clinicians should carefully weigh the potential benefits of sertraline against its adverse effects, taking into account individual patient characteristics such as age, sex, and duration of dialysis. Future studies, especially randomized controlled trials, are needed to focus on long-term safety, comprehensive adverse event reporting, and the exploration of subgroup differences to further refine the use of sertraline in this population.

## Conclusion

In conclusion, this meta-analysis supports the efficacy of sertraline in reducing depressive symptoms and improving the quality of life in dialysis patients with ESRD. While the increased risk of adverse events warrants caution, the overall benefits suggest that sertraline is a viable treatment option for this population. Studying the long-term effects of sertraline on dialysis patients is crucial for understanding its sustained efficacy and safety profile. Long-term studies should monitor patients over extended periods to evaluate the durability of antidepressant effects.

The key findings of our study underscore the importance of sertraline as a potentially effective treatment for depression in dialysis patients. Sertraline offers significant improvements in both depressive symptoms and quality of life measures. Clinicians should carefully weigh the efficacy of sertraline against its safety profile, considering individual patient characteristics, potential drug interactions, and individual patient characteristics such as age, sex, and duration of dialysis. Also, to gather sufficient data for a meta-regression analysis, several studies were conducted in partnership with dialysis centres and healthcare institutions to access a broader patient database. Future studies should also aim to incorporate objective measures of depressive symptoms by utilizing standardized clinical assessments conducted by trained professionals alongside self-reported questionnaires.

These results emphasize the need for further research, particularly with a larger sample size and more comprehensive multicentre studies across diverse geographical regions, to confirm the findings and optimize sertraline use in clinical practice. Further studies should focus on elucidating the long-term effects of sertraline, its impact on patient adherence to treatment, comprehensive adverse event reporting, the exploration of subgroup differences, and its overall cost-effectiveness.

## Ethical approval

Not applicable.

## Consent

Not applicable.

## Source of funding

The authors received no extramural funding for the study.

## Author contribution

All authors made a significant contribution to the work reported, whether that is in the conception, study design, execution, acquisition of data, analysis and interpretation, or all these areas; took part in drafting, revising or critically reviewing the article; gave final approval of the version to be published; have agreed on the journal to which the article has been submitted; and agree to be accountable for all aspects of the work.

## Conflicts of interest disclosure

The authors declare that they have no known competing financial interests or personal relationships that could have appeared to influence the work reported in this paper.

## Research registration unique identifying number (UIN)

We had applied for prospero registration at https://www.crd.york.ac.uk/prospero/ but it is not yet confirmed or accepted by the organization or provided us a number yet.

Acknowledgement receipt number (563162).

## Guarantor

Varsha Sharma.

## Data availability statement

The dataset supporting the conclusions of this article is included.

## Provenance and peer review

No it was not invited.

## Supplementary Material
