# Supplementary material for: Safety and efficacy of sertraline in depression among adults undergoing dialysis: a systematic review and meta-analysis
Source: Ann Med Surg (Lond). 2024 Oct 22;86(12):7082–93. doi: 10.1097/MS9.0000000000002677 (PMC11623899; doi:10.1097/MS9.0000000000002677)
Supplement: Supplementary file 1 [file ms9-86-7082-s001.docx]

**Supplementary Table 1.** Search Strategies for different Databases

| **Database** | **Search String** |
| --- | --- |
| PubMed/MEDLINE  (26 Results) | ("sertralin"[All Fields] OR "sertraline"[MeSH Terms] OR "sertraline"[All Fields] OR "sertraline s"[All Fields]) AND ("haemodialysis"[All Fields] OR "renal dialysis"[MeSH Terms] OR ("renal"[All Fields] AND "dialysis"[All Fields]) OR "renal dialysis"[All Fields] OR "hemodialysis"[All Fields]) AND ("depressed"[All Fields] OR "depression"[MeSH Terms] OR "depression"[All Fields] OR "depressions"[All Fields] OR "depression s"[All Fields] OR "depressive disorder"[MeSH Terms] OR ("depressive"[All Fields] AND "disorder"[All Fields]) OR "depressive disorder"[All Fields] OR "depressivity"[All Fields] OR "depressive"[All Fields] OR "depressively"[All Fields] OR "depressiveness"[All Fields] OR "depressives"[All Fields]) |
| Cochrane Library  (21 Results) | sertraline AND hemodialysis AND Depression |
| Science Direct  (563 Results) | sertraline AND hemodialysis AND Depression |
| Clinical Trial Govt  (2 results) | Intervention: sertraline; Condition: hemodialysis AND Depression |

**Supplementary Table 2.** Risk of Bias and its assessment.

|  | Cochrane Risk-of-Bias Tool | | |  |
| --- | --- | --- | --- | --- |
|  | Bias | Risk of bias | Author judgement | |
| FRIEDLI ET AL. 2017 | Random sequence generation (selection bias) | Low Risk | The patients were randomized using placebo vs sertraline on the basis of randomizing software. | |
|  | Allocation concealment (selection bias) | Low Risk | The author explicitly mentions that block randomization with stratification, the blinding of nurses, patients, and clinicians, and the blinding of clinicians further minimizes the risk of selection bias. | |
|  | Blinding of participants and personnel (performance bias) | Low Risk | It is a randomized, blinded study and placebo-controlled | |
|  | Blinding of outcome assessment (detection bias) | Low Risk | Blinding of the outcome assessor is acknowledged, which reduces the likelihood of detection bias. | |
|  | Incomplete outcome data (attrition bias) | Low Risk | Although there were dropouts from the sertraline group, it was handed properly, which minimizes the risk of attrition bias. | |
|  | Selective reporting (reporting bias) | Low Risk | There is no indication of selective reporting; the study has appropriately addressed the transparent reporting of outcomes and the screening process. | |
|  | Other bias | Low Risk | No other bias is reported in this study | |
| HEDAYATI ET AL. 2017 | Random sequence generation (selection bias) | Low Risk | The patients were randomized on 1:1 using a random number generator | |
|  | Allocation concealment (selection bias) | Low Risk | The author mentions the use of a computer-based random number generator, and participants and healthcare providers were blinded to the treatment allocation. | |
|  | Blinding of participants and personnel (performance bias) | Low Risk | It is a randomized, double-blinded, placebo-controlled parallel study. | |
|  | Blinding of outcome assessment (detection bias) | Low Risk | The study clearly mentioned that the outcomes were measured by the trained personnel who were blinded to treatment assignment. | |
|  | Incomplete outcome data (attrition bias) | Low Risk | There could be a risk of attrition bias because the author addressed the withdrawal of several participants during the trial, and few were lost during follow-up. However, the researcher has taken the appropriate measures to minimize the risk. | |
|  | Selective reporting (reporting bias) | Low Risk | There is no evidence of reporting bias in our study | |
|  | Other bias | Low Risk | No other biases were identified in the study that could significantly impact the results. | |
| MEHROTRA ET AL. 2019 | Random sequence generation (selection bias) | Low Risk | The study has two phases; each phase has a randomizing ratio of 1:1. Randomized selection of participants was done through a web portal. | |
|  | Allocation concealment (selection bias) | Low Risk | There is no potential risk of bias as the allocation of participants was completely hidden from the participants and researchers. | |
|  | Blinding of participants and personnel (performance bias) | high Risk | The study has not explicitly addressed the risk of performance bias. | |
|  | Blinding of outcome assessment (detection bias) | Low Risk | Outcome assessors were blinded to intervention allocation, reducing the risk of detection bias. | |
|  | Incomplete outcome data (attrition bias) | Low Risk | Incomplete outcome data were handled appropriately, minimizing the risk of attrition bias. | |
|  | Selective reporting (reporting bias) | Low Risk | There is no evidence of selective outcome reporting, reducing the risk of reporting bias. | |
|  | Other bias | Low Risk | No other biases were identified in the study that could significantly impact the results. | |
| ZHANG ET AL. 2024 | Random sequence generation (selection bias) | Low Risk | The participants were allocated randomly by using the software SPSS 26.0 with a ratio of 1:1 | |
|  | Allocation concealment (selection bias) | Low Risk | The allocation concealment was explicitly mentioned in the paper, which minimizes the risk of selection bias. | |
|  | Blinding of participants and personnel (performance bias) | unclear Risk | There is no mention of blinding of participants, and the risk remains unclear. | |
|  | Blinding of outcome assessment (detection bias) | Low Risk | Although the study does not mention blinding outcome assessors, the use of HAMD SCALE reduces the risk of detection bias. | |
|  | Incomplete outcome data (attrition bias) | High Risk | The study has reported the risk of attrition bias due to the high no of dropouts of participants during a trial. | |
|  | Selective reporting (reporting bias) | Low Risk | There is no evidence of selective outcome reporting; hence, it potentially decreases the risk of reporting bias. | |
|  | Other bias | Low Risk | No other biases were identified in the study that could significantly impact the results. | |
